# Histamine activates inflammatory response and depresses casein synthesis in mammary gland of dairy cows during SARA

**DOI:** 10.1186/s12917-018-1491-3

**Published:** 2018-05-23

**Authors:** Guangjun Chang, Lailai Wang, Nana Ma, Wenwen Zhang, Huanmin Zhang, Hongyu Dai, Xiangzhen Shen

**Affiliations:** College of Veterinary Medicine, Nanjing Agricultural University, Nanjing, Jiangsu China

**Keywords:** Mammary gland, Histamine receptor, NF-κB and mTOR signalling pathway, Casein, Inflammatory response

## Abstract

**Background:**

Mounting evidences observed that subacute ruminal acidosis (SARA) induced by high concentration (HC) diet increases the translocation of histamine from digestive tract into circulation causing a diverse of diseases in dairy cows. However, it is largely unknown how it does affect the function of mammary gland and milk quality. Hence, this study aims to observe the effects of histamine derived from the digestive tract on the inflammatory response and casein synthesis in the mammary glands during SARA. Twelve cows fitted rumen fistula were randomly divided into either control group administrated low concentration (LC) diet (60% forage, *n* = 6) or treatment group administrated HC diet (40% forage, *n* = 6) for 18 weeks.

**Results:**

Our data showed that HC diet resulted in significant declines in rumen pH value, milk yield and milk quality, as well as longer duration of averaged pH value below 5.6 per day (more than 180 min) compared to LC diet, these findings confirmed SARA occurence. Our study also observed that SARA increased the content of histamine in rumen fluid, plasma, liver and mammary gland, and enhanced the mRNA expression of histamine specific receptor in the mammary gland. Additionally, we found that the mRNA expression of inflammatory response genes in mammary glands was increased, which was consistent with the protein expression results, showing that the protein kinase C(PKC) / nuclear factor kappa B (NF-κB) or protein kinase A (PKA) / NF-κB signalling pathways of the inflammatory response were activated. The mRNA expression of mTOR, P70S6K and αS1 in mammary glands were significantly decreased with the protein expression of mTOR, P70S6K and αS1-casein, and the phosphorylation levels of the mTOR and P70S6K proteins were also decreased.

**Conclusions:**

Our study showed that the milk protein of lactating cows is depressed after long-term feeding of HC at the individual level, which was paralleled at the gene and protein levels. The inflammatory response in mammary gland caused by histamine derived from the digestive tract is related to the decline of casein synthesis. Our findings point to a new link between the inflammatory response and casein synthesis, but the understanding of the molecular mechanisms involved in this process will require further research.

**Electronic supplementary material:**

The online version of this article (10.1186/s12917-018-1491-3) contains supplementary material, which is available to authorized users.

## Background

Subacute ruminal acidosis (SARA) that is a common and frequent metabolic disorder disease can bring tremendous economic losses to dairy industry. The most important reason for SARA occurrence is that to maintain a high yield of milk, the nutrient density of dairy cow diets has increased [1]. The adjustment of the nutrient density is mainly through the feeding of HC diets. This feeding mode can increase the production of organic acids and cause a depression of the pH in the digestive tract over time [2, 3]. When the rumen pH value of cows falls below 5.6 for more than 3 h per day, SARA occurs. This decrease in pH may result in alterations in the type of fermentation and the composition of the microbiota in the rumen [4]. When the rumen has a low pH, the different types of bacteria make histidine for decarboxylization to produce histamine [5]. Histamine that is absorbed from the gastrointestinal tract can be rapidly detoxified by amine oxidases in healthy subjects, and it also can be produced by epithelial cell to regulate many physiological processes, such as cell growth and differentiation [6]. The acid-induced impairment of epithelial barrier function facilitates the translocation of histamine from the digestive tract into the bloodstream [7]. The increase of histamine in blood can cause systemic histaminosis such as laminitis, which is a popular dieses in dairy farm [1], and it is also closely associated with inflammation and lactation of mammary gland [6, 8], those observations suggested that histamine originated from gastrointestinal tract entering mammary gland may elicits local inflammation, and then affects lactation of mammary gland and milk quality.

Histamine (2-[4-imodazole]-ethylamine, HIS) is synthesized by the decarboxylation of histidine by L-histidine decarboxylase (HDC), which is dependent on the cofactor pyridoxal-5′-phosphate [9]. After histamine interacts with its receptors (H_s_R), of which there are four subtypes (H_1_R, H_2_R, H_3_R and H_4_R) that all belong to the family of G protein-coupled receptors, it will exert many (patho-) physiological effects [10]. H_1_R-coupled Gα_q/11_ can generate inositol-1, 4, 5-triphosphate and diacylglycerol and increase the intracellular calcium content through stimulating the inositol phospholipid signalling pathways, which has been demonstrated to activate NF-κB [11, 12]. H_2_R-coupled Gα_s_ can, through separate guanosine triphosphate-dependent mechanisms, activate adenylate cyclase and the phosphoinositide second messenger-cAMP to produce gastric acid and mucus [13, 14]. H_3_R is mainly present in nervous system signal transduction, and it can lead to the inhibition of cAMP, the accumulation of Ca^2+^ and the activation of the mitogen-activated protein kinase pathway to release neurotransmitters when it is coupled with Gα_i/o_ [15]. H_4_R has been reported to couple with Gα_i/o_ and play an important role in binding sites for interferon regulatory factor and NF-κB in the modulation of the immune system [16, 17].

Milk is a mixture of several ingredients, including water, fat, protein, lactose and inorganic salt. Meanwhile, casein is a complex particle containing calcium and phosphateand accounts for 80%~ 82% of the total amount of protein in milk. Casein has the following four groups: αS1-casein, αS2-casein, β-casein and κ-casein [18]. In dairy cows, the most abundantly expressed milk protein is αS1- casein. Janus kinase (JAK), the signal transducer and activator of transcription (STAT) signalling pathway and the mTOR signalling pathway are the primary pathways in milk protein expression. However, it has been reported that the role of the JAK-STAT signalling pathway is weak in regulating bovine milk protein expression [19], because the synthesis of αS1-casein is translational but not transcriptionally regulated. Previous studies have demonstrated that histamine can induce inflammation; however, it is unknown whether histamine derived from the digestive tract during SARA is capable of influencing inflammatory responses and casein synthesis in the mammary gland of dairy cows. Therefore, we hypothesized that the present study could investigate the effects of histamine derived from the digestive tract during SARA on the inflammatory response and casein synthesis in the mammary gland of lactating dairy cows fed high concentration (HC) diets.

## Methods

All experimental procedures were approved by the Animal Experiment Committee of Nanjing Agricultural University, in accordance with the Regulations for the Administration of Affairs Concerning Experimental Animals (The State Science and Technology Commission of China, 1988). All the experimental protocols were performed in accordance with the approved guidelines and regulations.

### Animals, diets, and experimental design

Twelve multiparous lactating Holstein cows were housed in individual tie- stalls in dairy farm of Nanjing Agricultural University and fitted with a rumen fistula (3–5 weeks post-partum, aged 3–5 years and averaged body weight 460 ± 25 kg) displayed an averaged milk yield 29 ± 3.17 kg/day at the beginning and 27 ± 2.76 kg/day at the end of the present experiment. All cows were fed low concentration (LC) diet for 1 month to reach a similar metabolic state, and then randomly divided into two groups. One group (*n* = 6) was administrated HC diet comprising 60% concentrate and 40% forage as treatment group, while another group (*n* = 6) was fed LC diet comprising 40% concentrate and 60% forage as control group. The experiment was lasted for 18 weeks. The details of the dietary ingredients and nutritional composition are presented in Table 1. Cows had fresh water for free drinking and were milked and fed at 04:00, 12:00, and 20:00 during the experiment.

**Table 1:** Ingredients of low concentrate and high concentrate diet

Item	Ingredient	LC	HC
Forage	Corn silage	30.00	20.00
	Alfalfa hay	30.00	20.00
Concentrate	Corn	22.78	33.60
	Bran	5.15	15.00
	Soybean meal	9.80	9.00
	Calcium phosphate dibasic	0.92	0.53
	Limestone	0.00	0.52
	Salt	0.35	0.35
	Premix^a^	1.00	1.00
	Total	100.00	100.00
Nutrient composition	NE(MJ/Kg)	6.32	6.74
	CP (%)	16.00	16.20
	EE (%)	3.96	4.15
	NDF (%)	37.71	31.92
	ADF (%)	22.75	17.55
	NFC (%)	33.43	40.31
	Starch (%)	25.33	32.28
	Ca (%)	0.90	0.80
	P (%)	0.45	0.45

^a^Premix contained VA 1900 ku/kg; VD 250 ku/kg; VE 3000 mg/kg; Niacin 4000 mg/kg; Cu 1200 mg/kg; Fe 525 mg/kg; Zn 13,000 mg/kg; Mn 5500 mg/kg; I 170 mg/kg; Co 50 mg/kg; Se 27 mg/kg

*LC* low concentration diet, *HC* high concentration diet

### Rumen pH and milk measurements

The pH value was measured by a pH-meterat1 h intervals for 24 h on the last 3 days of 18th week. Milk yield was measured at each of the 3 milkings on experimental days of the 1^st^week and 18th week. Milk sample (50 mL) was also taken from the mixture of 3 milkings of each cow on these days, and then it was added potassium dichromate and stored at 4 °Cfor the measurement of milk protein and fat with the Milko Scan™ FT1device (Foss, Denmark). At 4 h after feeding on the last day of 18th week, Rumen content and fluid were mixed, and then the mixture was taken via rumen fistula, and squeezed to obtain rumen fluid (50 mL), which subsequently was filtered by four layer gauze. The filtrate was centrifuged at 10000×g for 30 min, and then the supernatant was stored at − 20 °C for further histamine determination.

### Tissue and blood samples collection

On the last 3 days of the 18thweek, Blood were collected 4 h after feeding via the lacteal artery into 5-ml heparinised vacuum tubes, and plasma was isolated by centrifugation at 1900×g for 15 min and stored at − 20 °C for histamine determination. Approximate 10 g of Mammary gland tissue of each cow was harvested by biopsy from a deeper location of the right hind quarter. One small portion (about 0.5 g) supplemented with phosphate buffer saline (PBS, 0.5 mL) was homogenized using a Dounce homogenizer and centrifuged at 3000×g for 10 min, and then the supernatant was stored at − 20 °C for histamine determination. Another portion of mammary gland tissue was stored at − 80 °C.After sampling the mammary gland, the cows were slaughtered, and the liver tissue was collected aseptically within 5 to 10 min after killing, then liver tissue samples were cut into several smaller tissue pieces (about 0.5 g), which was prepared following the processing protocol of mammary gland for histamine determination and stored at. -20 °C.

### Enzyme-linked immunosorbent assay

Histamine concentrations in rumen fluid, plasma, liver and mammary gland tissue were determined with a double-antibody sandwich enzyme-linked immunosorbent assay (cat.H171, JIANCHENG, China). According to the instruction of manufacturer, diluted histamine standard (1600 ng/mL to 100 ng/mL) and prepared samples was added to the monoclonal antibody enzyme well, which was pre-coated with bovine HIS monoclonal antibody, followed by incubation. Then, histamine antibody labelled with biotin was added and combined with Streptavidin-HRP to form an immune complex. The plate was incubated and washed 5 times to remove the uncombined enzyme. After the addition of Chromogen Solution A and B, the plate was read by the Microplate Reader (Bio-TekELX80, USA) to obtain the optical density value (OD value). The standard curve was made according to the OD value and corresponding concentration. Histamine concentration in the samples was calculated by the standard curve.

### RNA extraction, cDNA synthesis and real-time polymerase chain reaction (PCR)

Total RNA was extracted using TRIzol following the instructions of the kit (cat.9108, Takara, Dalian, China). The concentration and the quality of the RNA were detected using a NanoDrop ND-1000 Spectrophotometer (ThermoFisher Scientific Inc., Waltham, USA). 400 ng of the total RNA was used to synthesize cDNA through the PrimeScript RT Master Mix Perfect Real Time kit (cat.RR036A, Takara, Dalian, China). The real-time PCR was conducted using the SYBR Premix Ex TaqTM kit (cat.DRR420A, Takara, Dalian, China) on an ABI 7300 Real-Time PCR System (Applied Biosystems, Foster City, CA, USA). The PCR protocol was denaturation at 95 °C for 15 s, then 40 cycles at 95 °C for 5 s, and 60 °C for 31 s. Glyceraldehyde phosphate dehydrogenase (GAPDH) was used as the internal reference gene. The primers were designed using Primer Premier Software 5.0 (Premier Biosoft International, USA) and synthesized by Generay Company (Shanghai, China), and the primer details are shown in Table 2. The 2^-△△^Ct method was used to analyse the results.

**Table 2:** The primer list for reverse transcription and amplification of RT-qPCR

*Gene*	*NCBI Accession*	*PCR product*	*Forward Primer (5’-3’)*	*Reverse Primer (5′ -3’)*
H_1_R	NM_174083.4	245 *bp*	AGCCAGAACCAGCTTGAGAT	TTCATGTGCAAGCCAGACAC
H_2_R	XM_015464941.1	198 *bp*	TTGGCAAGGTCTTCTGCAAC	GGAAGGACAGGGTGATGGAA
H_3_R	XM_015466045.1	172 *bp*	CAGAAGATGGTGCTGGTGTG	AAGGGGTGAAGAACTCGAGG
H_4_R	XM_001251983.5	221 *bp*	ATCCTTGCCCTCACGTTAGT	CGAAGGGATGCTGCTGATTC
IL-1β	NM_174093.1	167 *bp*	GGCCAAAGTCCCTGACCTCT	CTGCCACCATCACCACATTC
TNF-α	NM_173966.3	261 *bp*	CACATACCCTGCCACAAGGC	CTGGGGACTGCTCTTCCCTCT
NF-κB	NM_001045868.1	129 *bp*	ATACGTCGGCCGTGTCTAT	GGAACTGTGATCCGTGTAG
mTOR	XM_001788228.1	199 *bp*	ATGCTGTCCCTGGTCCTTATG	GGGTCAGAGAGTGGCCTTCAA
P70S6K	NM_205816.1	162 *bp*	GGACATGGCAGGGGTGTTT	GGTATTTGCTCCTGTTACTTTTCG
4EBP1	BC120290.1	177 *bp*	GGCAGGCAGTGAAGAGTC	CCTGGGCTGCGGGAT
αS1-casein	NM_181029.2	170 *bp*	ATTTTCAGACAATTCTACCAGCT	AATTCACTTGACTCCTCACCAC
GAPDH	NM_001034034.2	135 *bp*	TGTTGTGGATCTGACCTGCC	AAGTCGCAGGAGACAACCTG

### Western blotting

Total protein was extracted from frozen mammary gland tissues. One hundred mg of frozen minced mammary tissues and 1 mL ice-cold RIPA lysis buffer (cat. SN338, Sunshine Biotechnology Co., Ltd., Shanghai, China) were mixed and homogenized using a Dounce homogenizer. The supernatant was collected after centrifugation at 12,000 rpm for 15 min at 4 °C. The protein concentration was measured using a BCA Protein Assay kit (cat.23225, ThermoFisher, USA) and then diluted to the same concentration. Fifty μg of protein from each sample was loaded onto 8~ 12% sodium dodecyl sulphate polyacrylamide gel electrophoresis (SDS-PAGE) gels. After separation, the proteins were transferred to a nitrocellulose (NC) membrane (Pall Gelman Laboratory, USA). The membrane was blocked with 6~ 7% nonfat dry milk (dissolved in Tris-Buffered Saline with Tween, TBST) or 6~ 7% BSA (for phosphorylated protein) for 2 h at room temperature. Afterwards, the NC membrane was washed and incubated with TBST-diluted primary antibody at 4 °C overnight. The primary antibodies were used in previous study [20], and diluted at the following ratios: NF-κB p65 (1:500;cat.AN365, Beyotime, China), phosphorylated NF-κB p65 (1:500; cat.AN371, Beyotime, China), IL-6 (1:200; cat.sc-1265, Santa Cruz, USA), GAPDH (1:5000;cat.AP0066, Bioworld, USA), αS1-casein (1:400; cat.sc-376,961, Santa Cruz, USA), mTOR (1:500; cat.sc-8319, Santa Cruz, USA), phosphorylated mTOR (1:500; cat.sc-101,738, Santa Cruz, USA), P70S6K (1:500; cat.sc-9027, Santa Cruz, USA) and phosphorylated P70S6K (1:500; cat.sc-7984R, Santa Cruz, USA). The membranes were then washed and incubated with the corresponding secondary antibodies for 2 h at room temperature. The secondary antibodies were as follows: goat anti-rabbit (1:10000; cat.sc-2004, Santa Cruz, USA) for NF-κBp65, NF-κBpp65, IL-1β, P70S6K, mTOR, pP70S6K, and pmTOR; goat anti-mouse (1:10000; cat.sc-2005, Santa Cruz, USA) for αS1-casein; rabbit anti-goat (1:10000; cat.sc-2768, Santa Cruz, USA) for GAPDH. Finally, the membranes were treated with an enhanced chemiluminescence (ECL) detecting kit (E411–04, Vazyme Biotech Co.,Ltd., Nanjing, China). The signals were recorded through an imaging system (LAS4000, USA), and the results were analysed using Quantity One software (Bio-Rad, USA).

### Statistical analysis

Data from milk and rumen pH was analyzed using repeated model of MIXED procedure of SAS (SAS version 9.4, SAS Institute Inc.). The effects of diet and days were considered as fixed factors, and goat was treated as random factor. Days with diet and goat was considered as repeated measure, and compound symmetry (CS) was used as the type of covariance. Data about histamine concentration, mRNA and protein expression was analyzed using ANOVA procedure of SAS. The significant difference was considered to occur when *P* < 0.05.

## Results

### Ruminal pH, milk yield and milk composition

Compared with LC diet, HC diet was significantly decreased the mean rumen pH value and increased the duration of rumen pH < 5.6/day, which was longer than 180 min per day after 18^th^ week feeding.(Table 3).Additionally, the feeding of HC diet caused an increased tendency in milk yield and significant increase in milk fat in the 1st week, whereas resulted in significant decline in milk yield, milk protein and milk fat in the 18^th^week compared to the feeding of LC diet.

**Table 3:** Rumen pH, milk yield and composition of dairy cows fed LC or HC diets

Item	LC	HC	SEM	*p*-Value		
				Diet	Day	Diet × Day
Mean pH value	6.02	5.90	0.03	< 0.01	0.23	0.18
Minimum pH value	5.65	5.50	0.05	0.02	0.08	0.38
Time < pH 5.6, min/d	99.00	223.00	30.25	0.01	0.06	0. 57
Milk yield (kg/d) week1	28.7	30.9	0.47	0.08	0.23	0.76
Milk yield (kg/d) week18	28.05	26.92	0.30	< 0.01	0.05	0.41
Milk protein (%) week1	3.34	3.41	0.03	0.11	0.53	0.79
Milk protein (%) week18	3.02	2.88	0.04	0.01	0.51	0.31
Milk fat (%) week1	3.48	3.34	0.02	0.21	0.61	0.58
Milk fat (%) week18	3.54	2.94	0.04	0.02	0.12	0.81

### Histamine content in rumen fluid, blood, liver tissues and mammary gland tissues in dairy cows

**Fig. 1:**
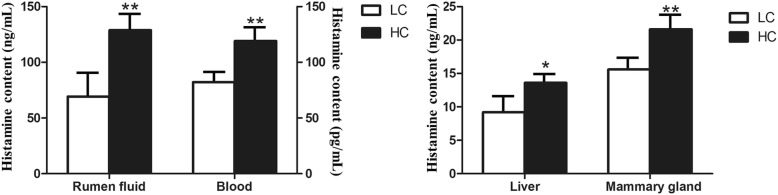
Histamine concentrations in rumen, blood, liver and mammary tissues in the LC and HC group. The ordinate axis indicates the absolute histamine content in tissue (*n* = 6 in each group, mean ± SEM). The white packed column represents the LC group, while the black packed column represents the HC group. The significant differences are indicated (*P < 0.05, **P < 0.01)

The mean histamine values in rumen fluid, blood, liver tissue and mammary gland tissue were measured and the results presented in Fig. 1 show that the values in the four tissues were significantly different between the LC and HC groups. The histamine content in the rumen fluid and blood of the HC group was significantly increased (*P* < 0.01; Fig. 1a) compared to the LC group. The histamine content in the liver tissues was higher compared with the liver tissues, and the differences in the liver tissues and mammary tissues between the LC and HC group were both significant (*P* < 0.01; Fig. 1b).

### mRNA expression of histamine receptors in mammary gland tissues

**Fig. 2:**
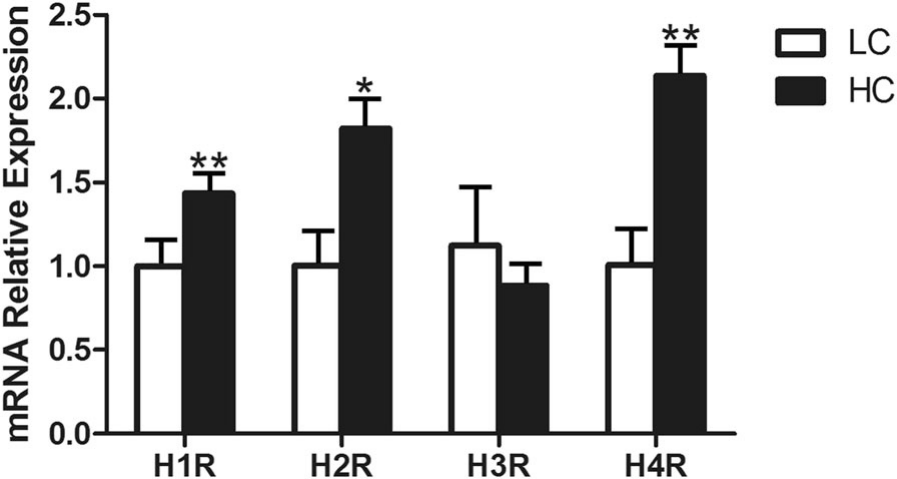
mRNA expressions of the histamine receptor genes H_1_R, H_2_R, H_3_R and H_4_R in the mammary tissues of dairy cows in the LC and HC groups. The ordinate axis indicates the relative expression of histamine receptors in mammary gland tissues (*n* = 6 in each group, mean ± SEM). The white packed column represents the LC group, while the black packed column represents the HC group. The significant differences are indicated (*P < 0.05, **P < 0.01)

The results showed that the different types of histamine receptors in the mammary gland tissues had heterogeneous expression, of which H_4_R had the highest expression, while the expression of H_3_R was the lowest (Fig. 2). H_1_R, H_2_R and H_4_R had significantly different expressions in the LC group and HC group (*P* < 0.01). There was not a significant difference in H_3_R expression between the LC and HC groups.

### mRNA expression of inflammatory response genes in mammary gland tissues

**Fig. 3:**
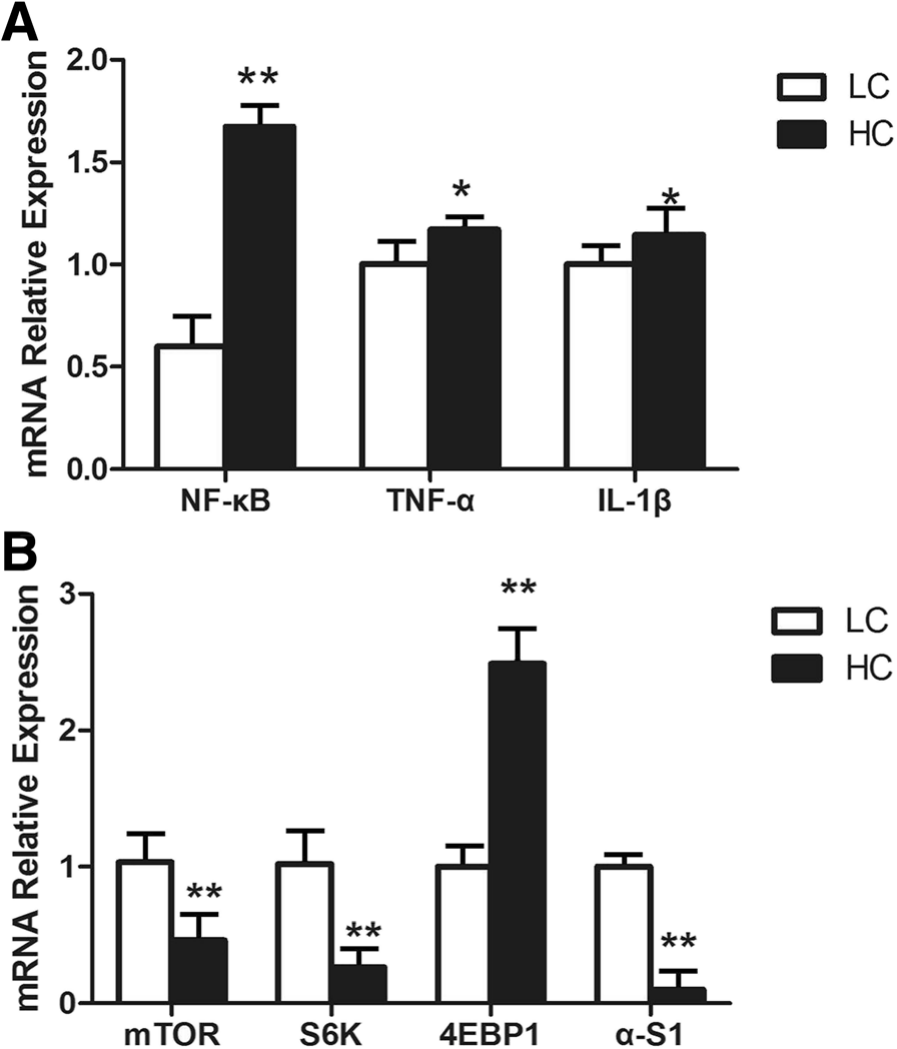
mRNA expressions of inflammatory genes and casein synthesis in the mammary glands of dairy cows in the LC and HC groups. **a** shows the mRNA expression of inflammatory response genes in mammary gland tissue, while (**b**) shows the mRNA expression of casein synthesis genes. The ordinate axis indicates the relative expression of the genes in mammary gland tissues (*n* = 6 in each group, mean ± SEM). The white packed column represents the LC group, while the black packed column represents the HC group. The significant differences are indicated (*P < 0.05, **P < 0.01)

The results showed that NF-κB mRNA expression in mammary tissues in the HC group was significantly high (*P* < 0.01) compared to the LC group (Fig. 3a). mRNA expression of tumour necrosis factor (TNF)-α and interleukin(IL)-1β were significantly up-regulated in the HC group compared with the LC group (*P* < 0.05).

### mRNA expression of casein synthesis genes in mammary gland tissues

As shown in Fig. 3b, the mRNA expression of casein synthesis genes was changed by HC feeding. The mRNA expression of mTOR genes was significantly decreased in the HC group in mammary gland tissues compared with the LC group (*P* < 0.01). The mRNA expression of the S6K genes was significantly decreased in the HC group, but the mRNA expression of the 4EBP1 genes was markedly increased (*P* < 0.01) in the HC group. Importantly, the αS1-casein genes in the HC group had significantly decreased mRNA expression compared with the LC group in mammary gland tissues.

### Protein expression of inflammatory factors in mammary gland tissues

**Fig. 4:**
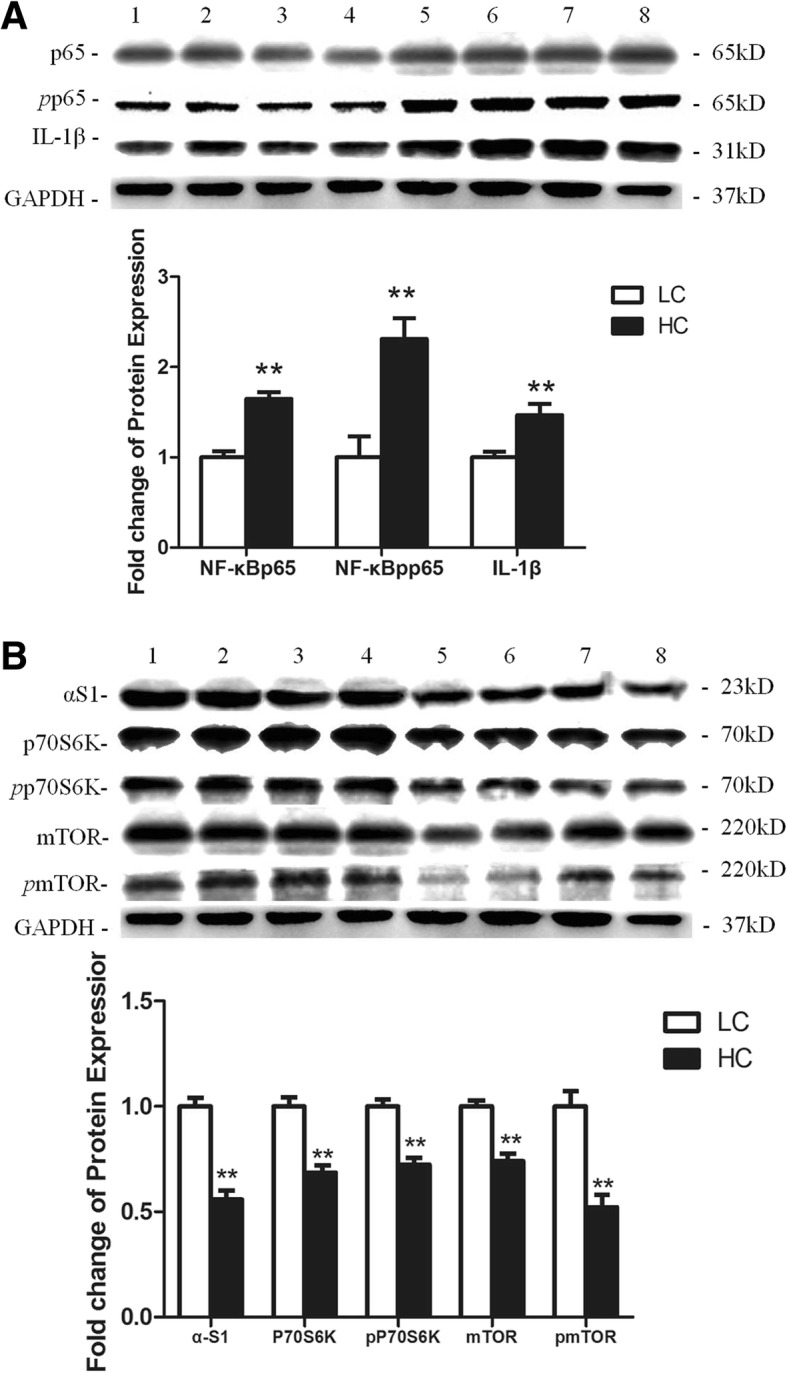
Protein expressions of several inflammatory factors and casein synthesis in the mammary glands of dairy cows in the LC and HC groups. **a** shows the protein expression of several inflammatory factors in mammary gland tissue, while (**b**) shows expression of casein synthesis proteins in the mammary glands of dairy cows in the LC and HC groups. The samples used for western blot derived from the same experiment and the blots for target proteins were conducted in parallel. GAPDH was used as an internal reference protein, and the band density was used to quantify protein expression. The cropped bands 1–4 bands represent the LC group, while 5–8 bands represent the HC group. The significant differences are indicated (**P < 0.01)

Western blotting results (Fig. 4a) showed that the NF-κBp65 and NF-κB *p*p65protein in mammary glands were both significantly increased (*P* < 0.01) in the HC group. The significant increase of IL-1β (*P* < 0.01) further demonstrated the inflammation of the mammary gland tissues in the HC group.

### Protein expression of casein synthesis in mammary gland tissues

Western blotting results (Fig. 4b) showed that the expression of the αS1-casein, P70S6K, and mTOR proteins in the mammary glands were all decreased in the HC group compared with the LC group (*P* < 0.01). Meanwhile, the phosphorylated proteins *p*P70S6K and *p*mTOR were both down-regulated in the HC group compared with the LC group (*P* < 0.01). The changes of protein levels were consistent with the change in gene expressions. The original western blotting bands for each target protein were shown in Additional file 1.

## Discussion

SARA effects are caused by a combination of ruminal pH and diet type [21]. The pH results of the present study demonstrated that the dairy cows had experimentally SARA at the end of experiment through the feeding of an HC diet for 18 weeks, as the defined by the definition of previous study [22]. When SARA occurs, the histamine produced by rumen bacteria can easily pass through the damaged rumen epithelium into the bloodstream and cause inflammation [23, 24].

In fact, the concentrations of histamine in the rumen and plasma both increase during SARA. However, the absorption of histamine in the rumen is the direct cause of the increase in the concentration of histamine in the blood. Under normal conditions, dairy cows contain trace amounts of histamine [23], and when histamine is synthesized, it usually combines with polysaccharide sulphate, heparin and chondroitin sulphate to form inactive complexes stored in mast cell granules. The trace amount of histamine generally undergoes methylation or oxidation in the liver, where it is rapidly converted to a non-active substance in the urine. The results of the present study found that the histamine content is higher in the tissue of the mammary gland than in the tissue of the liver, which may be related to liver clearance. Meanwhile, the results illustrated that the liver has a higher tolerance to histamine than the mammary gland, as the mammary gland is more susceptible to the level of histamine [25].

Histamine can cause inflammation through the NF-κB signalling pathway, and this function is realized through combination with its receptors [26]. H_1_R-coupledGαq_/11_, H_3_R-coupled Gα_i/o_ and H_4_R-coupled Gα_i/o_ can activate PKC and H_2_R-coupled Gα_s_ can activate PKA, both of which can further activate NF-κB through the classical pathway or the alternative pathway [27]. Most inflammatory genes contain NF-κB binding sites within their promoters, and therefore once NF-κB has been activated, it will lead to the secretion of proinflammatory cytokines such as TNF-α and IL-1β [28, 29]. Our research found that three histamine receptors, H_1_R, H_2_R and H_4_R, were significantly increased during SARA, which may be closely related to their function. Histamine activation of H_1_R can change vascular permeability by acting on vascular endothelial cells and causing contraction of vascular smooth muscle cells; these features may exacerbate the inflammatory response [30]. Meanwhile, the expression of H_1_R genes can be up-regulated by histamine [31]. Compared with H_1_R, the absence of histamine can down-regulate the expression of H_2_R genes in a tissue-specific manner [32]. However, unlike H_1_R, histamine activation of H_2_R has effects on the relaxation of smooth muscle cells in the blood vessels and inhibition of cytokine production [33]. Recent work has highlighted the major role of H_4_R in eosinophils, mast cells, neutrophils, T-cells and dendritic cells as well as the production of cytokines influenced by these cells, which confirms the importance of this receptor in inflammatory responses [34–37]. We also found that H_3_R expression was not different between the LC and HC groups. According to previous studies, H_3_R is an auto-receptor regulating histamine release, and a knock-out of H_3_R in mice resulted in an increased severity of neuro-inflammatory diseases with enhanced cytokine release [38, 39]. The mRNA expression of NF-κB, TNF-α and IL-1β genes was significantly increased in the HC group, which was consistent with the results of the protein expression levels of NF-κBp65, NF-κBpp65 and IL-1β in the HC group. This showed that the PKC- NF-κB or PKA- NF-κB signalling pathway in the HC group was activated.

It has been reported that the phosphatidyl inositol 3 kinase(PI3K)-serine and threonine protein kinase(Akt)-mTOR signalling pathway plays a major role in casein synthesis. PI3K is activated to convert phosphoinositide 4, 5-bisphosphate (PIP2) into phosphoinositide 3, 4, 5-triphosphate (PIP3) [40]. Then, the growth factor activated kinase Akt is phosphorylated by the products of PI3K in the activation loop (Thr308) and recruited to the plasma membrane [41]. Accordingly, activated Akt can phosphorylate the negative regulator tuberous sclerosis protein-2 (TSC2) and dissociate the TSC complex into TSC1 and TSC2 through the lysosome [42]. Ras homologue enriched in brain (Rheb) is a small guanosine triphosphatase (GTPase) that can activate mTORC1 in its GTP-loaded state, while the GTPase-activating protein (GAP) activity of TSC2 hydrolyses Rheb-GTP into Rheb-GDP to inactivate mTORC1 [43]. The activated mTORC1 can phosphorylate protein translational regulators P70S6K and eukaryotic cells and translation initiation 4 e binding protein 1(4EBP1) [44]. Phosphorylated P70S6K can stimulate cell growth, while the function of 4EBP1 to stimulate protein translation is inhibited when it is phosphorylated. We found that the mRNA expression of mTOR, P70S6K and α-S1 was significantly decreased in the HC group, in agreement with the protein expression of mTOR, P70S6K and α-S1. Meanwhile, the phosphorylation level of mTOR and P70S6K protein in the HC group was also decreased. Collectively, the present study indicated that histamine derived from rumen caused the inflammatory response and reduction of casein synthesis in the mammary gland via activating the NF-κB and mTOR signalling pathway during SARA. It is well known SARA can cause diverse and complex consequences [1], and the present result is only one of consequences and may provide a new way to control inflammatory response of mammary gland elicited by SARA.

## Conclusion

Our research showed that the milk protein of lactating cows is depressed after long-term feeding of HC at the individual level, which was paralleled at the gene and protein levels. The inflammatory response caused by histamine derived from the rumen is related to reduce casein synthesis. Our findings suggest new relationships between the inflammatory response and casein synthesis, but the understanding of the molecular mechanisms involved in this process will require further research.

## Additional file

Additional file 1: Original western blotting band pictures. (DOCX 11282 kb)

## Abbreviations

HC: High concentration
IL-1β: Interleukin-1β
JAK: Janus kinase
LC: Low concentration
NF-κB: Nuclear factor kappa B
PKA: Protein kinase A
PKC: Protein kinase C
SARA: Subacute ruminal acidosis
STAT: The signal transducer and activator of transcription
TNF-α: Tumour necrosis factor-α
