# Supplementary material for: Histamine activates inflammatory response and depresses casein synthesis in mammary gland of dairy cows during SARA
Source: BMC Vet Res. 2018 May 23;14:168. doi: 10.1186/s12917-018-1491-3 (PMC5966854; doi:10.1186/s12917-018-1491-3)
Supplement: Supplementary file 1 — Original western blotting band pictures. (DOCX 11282 kb) [file 12917_2018_1491_MOESM1_ESM.docx]

Additional file 1 **Original western blotting band pictures**

p65





*p*p65


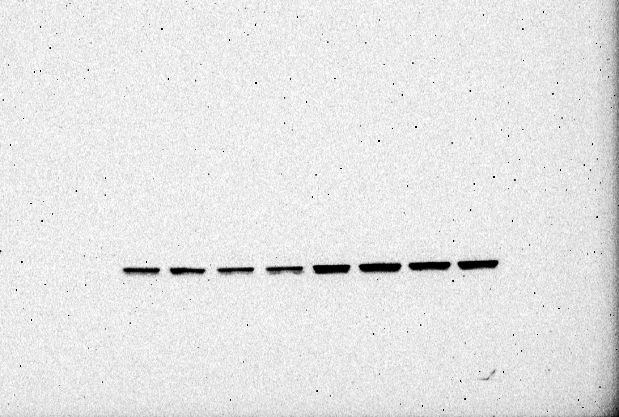


IL-1β





mTOR





αS1-casein


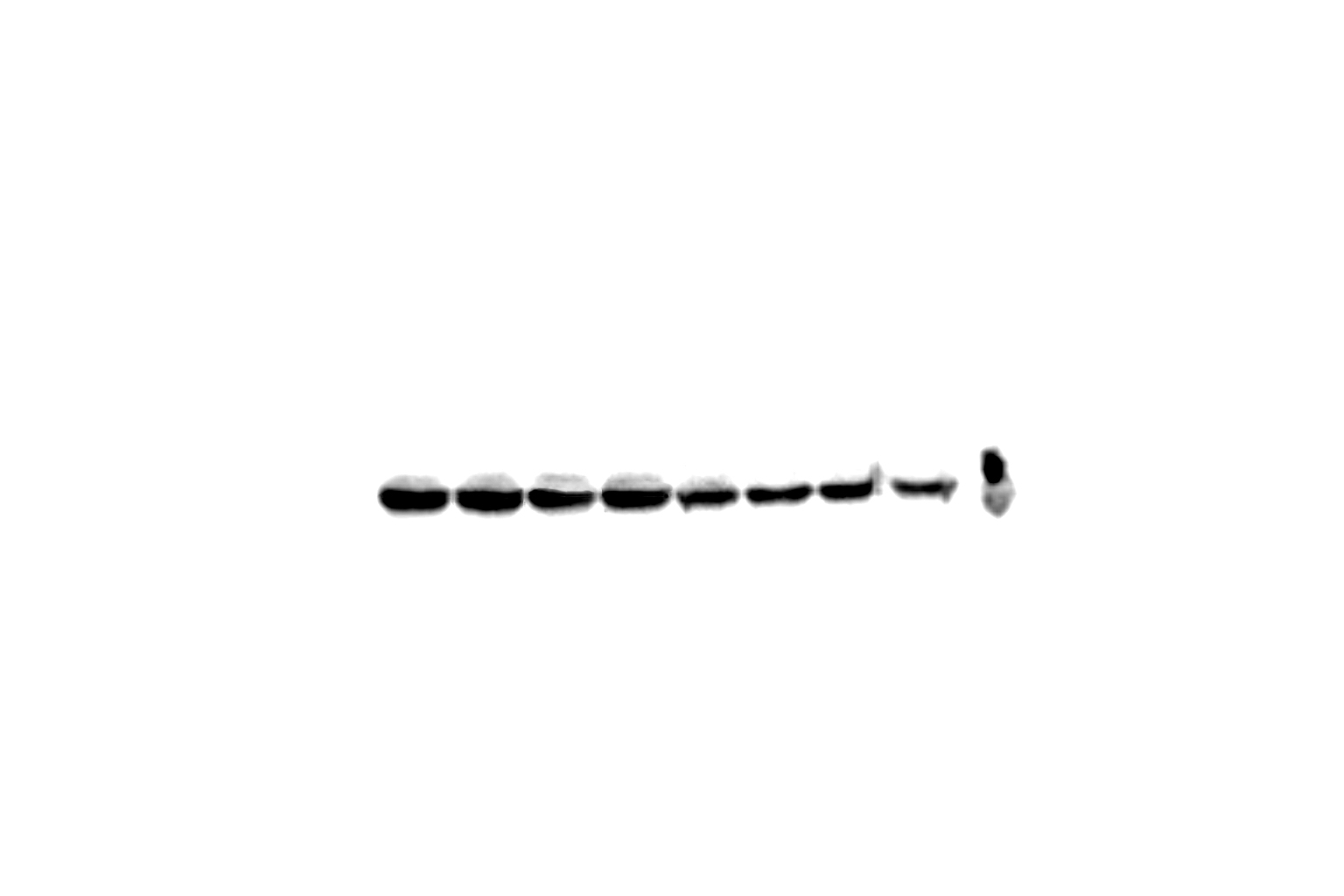


*p*mTOR





P70S6K





*p*P70S6K





GAPDH
